# Tracheobronchopathia osteochondroplastica: A rare cause of chronic cough with haemoptysis

**DOI:** 10.1186/1745-9974-4-4

**Published:** 2008-06-30

**Authors:** Hinrich Willms, Volker Wiechmann, Ulrich Sack, Adrian Gillissen

**Affiliations:** 1 Robert-Koch-Hospital, St. George Medical Center, Nikolai-Rumjanzew-Str. 100, D-4207 Leipzig, Germany; 2 Institute of Pathology and Tumour Diagnostic, St. George Medical Center, Delitzscher-Str. 141, D-04129 Leipzig, Germany; 3 Institute of Clinical Immunology and Transfusion Medicine, Medical Faculty of the University, Johannisallee 30, D-04103 Leipzig, Germany

## Abstract

A case of tracheobronchopathia osteochondroplastic (TPO) was diagnosed in a 69-year old male with prolonged cough. TPO is a rare condition of unknown cause and only sporadic cases have been reported. The condition is benign, characterized by submucosal nodules growing from the submucosal layer of the airways, protruding into the bronchial lumen. The bronchscopic view together with bronchial cartilage with abnormal distributed mineralization of the histologic examination of theses nodules leads to the correct diagnosis. Mild cases are treated symptomatically, whereas we tried an inhaled corticosteroid. Prominent protrusions in the trachea or the bronchi must be removed. In most cases the disease is stable over years but progressive forms have been reported. TPO may cause chronic refractory cough, which eventually is the only prominent symptom of this disease.

## Background

Tracheobronchopathia osteochondroplastica (TPO) is a rare benign disorder of the lower part of the trachea and the upper part of the main bronchi [1-3]. It was first described in the middle of the 19^th ^century and since than, approximately 300 cases have been reported. A higher incidence of TPO was seen in northern Europe countries, especially in Finland [4]. Because many cases are asymptomatic TPO is mainly diagnosed post mortem. Symptoms can range from productive or non-productive cough, haemoptysis, dyspnoea, dryness of the throat, recurrent pulmonary infections (e.g. retention pneumonia) or ozaena [4-7]. In severe cases the diagnosis is made during a difficult intubation [1,8]. The characteristic bronchoscopic finding is described as beaded, speculated, rock garden, cobble stoned or stalactite grotto appearance [9]. The diagnosis is confirmed by the typical histological appearance.

## Case presentation

A 69-year-old male presented to our pulmonary and critical care center suffering from chronic dry cough since several months and haemoptysis since about 4 weeks. Because of the cough and an assumed respiratory infection, he was treated with cefuroxim and moxifloxacine. Because lacking any apparent success, he finally was admitted to our center where he complaint about intermittent sweating at night, fever up to 39°C, and weight loss about 6 kg during the last 2 months. Total cigarette consumption was about 30 pack-years but he stopped smoking 25 years ago. History revealed no dust exposure. Allergies were unknown.

**Figure 1:**
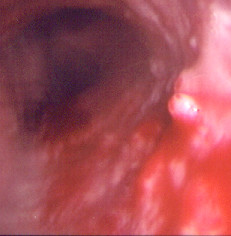
Bronchoscopic view of the trachea. Multiple tubercular nodules are seen (arrow).

**Figure 2:**
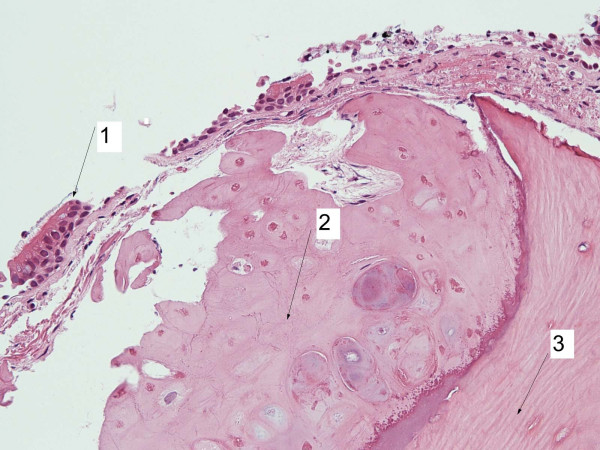
Bronchial cartilage with abnormal and unevenly distributed mineralization leads the diagnosis tracheobronchopathia osteochondroplastica: Tubercular nodule (haematoxylin-eosin x 50), normal bronchial epithelium (1), new cartilage (2) in abnormal submucous position with metaplastic ossification (3).

Apart from fine crackles over the lower part of both lungs physical examination was normal. Blood tests showed elevated c-reactive protein (41.0 mg/l), and slight anemia (erythrocytes: 4.05 Tpt/l; hemoglobin: 8.4 mmol/l) was apparent. Both chest X-ray and lung function tests were normal (VC 3.4 liters [76% predicted], FEV_1 _2,5 liters [76% predicted], FEV_1_/FVC 74%). Further, diffusing capacity and arterial blood gas values did not reveal any abnormalities. We first performed a gastroscopy, which turned out to be normal as well. Due to haemoptysis the patient underwent flexible fiber-optic bronchoscopy where we found in the middle of the trachea up to the main carina multiple tubercular nodules (Fig. 1). From these, various biopsies were taken, because we initially expect them to be malignant. In contrast, histological examination revealed bronchial cartilage and lamellar bone with little marrow (Fig. 2), a clear evidence for TPO. The mucous membrane of the trachea was lumpy, stiff and bled easily. Secretion was copious. Cytologic brushes of the trachea wall revealed bronchus epithelium with an accumulation of neutrophils. Smears and cultures for Mycobacterium tuberculosis were all negative. To reduce the inflammatory process of the trachea, and thus treating the cough [10], the patient was treated with inhaled budesonide (2 × 200 μg/day), and he eventually was dismissed from the hospital.

## Discussion

Sometimes TPO is diagnosed in a routine bronchoscopy, or it is seen coincidently in CT-scan or MRI [11-13,8]. Until now, approximately 300 cases worldwide have been reported. In our center with ca. 2 500 bronchoscopies/year, it was the first case in 10 years. There seems to be a higher prevalence in northern Europe, especially in Finland from which about 25% of all cases have been reported [4]. Cold-air-related hyperreactivity of the airway epithelium, high incidence of respiratory infection due to the cold climate together with a predisposing genetic factor or simply higher awareness by the doctors were discussed to be contribute factors [14]. But other contributing factors may be possible, because an association of habitually isolated M. ozaenae indicate that chronic infections with this bacterium and/or other germs may have a promoting effect although the exact mechanism is unknown [5,15]. Reduction of mucociliary transport, metaplasia of the connective tissue, exostosis arising in the cartilaginous ring, chronic inflammation with a possible link to amyloidosis of the lung are currently the most frequent hypothesis how TPO develops on the cellular level [9,2,4]. Once the disease is rare, it seems impossible to prove these hypotheses in a controlled trial. No gender predominance has been reported. Although most patients are older than 50 years, TPO is also found in children [16].

In the bronchoscopic view TPO appears as whitish, hard spicules projecting into the tracheal lumen from the anterior and lateral walls, with sparing of the posterior wall. Also the larynx and the main bronchi could be involved [17,6]. The diagnosis TPO is confirmed by typical histological findings, usually from biopsies or post mortem analysis. In severe cases CT scan reveals spicules in the trachea when they are big [11]. Our case was comparably mild because the small whitish nodules occurred mainly in the distal two thirds of the trachea which did not obstruct the lumen. Consequently, our patient did not suffer from dyspnea or asthma like symptoms like sever cases reported in the literature. The chronic cough is most likely caused be TPO because we did not find other causes although the patient underwent rigorous diagnostic procedures.

Besides of TPO nodules may also be caused by endobronchial sarcoidosis, calcificating lesions of tuberculosis, papillomatosis, malignant lesions and tracheobronchial calcinosis [4,9]. Some patients were initially thought to have asthma [18] or bronchial/trachea tumors like in our case or a middle lobe syndrome [11].

Because typical symptoms are absent, TPO is most likely under diagnosed. Only severe cases suffer from wheezing and dyspnoea caused by the obstruction of the airway lumen. Sometimes TPO causes difficulties in endotracheal intubation [12,17]. In most cases the disease progresses very slowly although progression have been reported eventually leading to respiratory insufficiency [8,19]. Once no specific therapy is available treatment is only symptomatic, which includes antibiotics in case of bacterial infections, mechanical measures to remove obstruction nodules using either cryotherapy, laser excision, external beam irradiation, radiotherapy, stent insertion or surgical resection therapy [20,12,3].

In conclusion, patients with chronic cough must undergo bronchoscopy at some time in order to uncover the underlying cause which may be a rare disorder [13,21].

## Consent

Written informed consent was obtained from the patient for publication of this case report and any accompanying images. A copy of the written consent is available for review by the Editor-in-Chef of this journal

## Competing interests

The authors declare that they have no competing interests.

## Authors' contributions

HW worked with the patient and did all the clinical work for diagnostics and therapy. Further, he wrote the first draft of the manuscript, VW evaluated the biopsies taken from our patient and prepared the histologic figure, US was involved in drafting the manuscript. He suggested sending it to "Cough", and he revised every manuscript version meticulously, AG wrote the manuscript based on the first version of HW. He further did all revisions of the manuscript, including the numerous suggestions made by US
